# Precision-cut liver slices: a versatile tool to advance liver research

**DOI:** 10.1007/s12072-018-9913-7

**Published:** 2018-12-04

**Authors:** Elena Palma, Ewald Jan Doornebal, Shilpa Chokshi

**Affiliations:** 1 Institute of Hepatology London, Foundation for Liver Research, London, UK; 2 Faculty of Life Sciences and Medicine, King’s College London, London, UK

**Keywords:** Liver slices, Organotypic system, Liver disease model

## Abstract

**Abstract:**

Human precision-cut liver slices represent a robust and versatile ex vivo model which retains the complex and multi-cellular histoarchitecture of the hepatic environment. As such, they represent an ideal model to investigate the mechanisms of liver injury and for the identification of novel therapeutic targets.

**Graphical abstract:**

Schematic overview to highlight the utility of precision-cut liver slices as a relevant and versatile ex-vivo model of liver disease. Top panel; Precision cut liver slices (PCLS) exposed to ethanol develop mega-mitochondria, a classical hallmark of Alcoholic Liver Disease (ALD). Right panel; PCLS from liver tumours can be used as a model for liver cancer and can be used to investigate cancer-immune cell interactions by co-culturing with matched immune cells. Bottom panel; Exposure to a mixture of oleic and linoleic acids can simulate Non-Alcoholic Fatty Liver Disease (NAFLD). Left panel; PCLS can be infected with Hepatitis B and C virus and used as a model to study viral infection and replication.
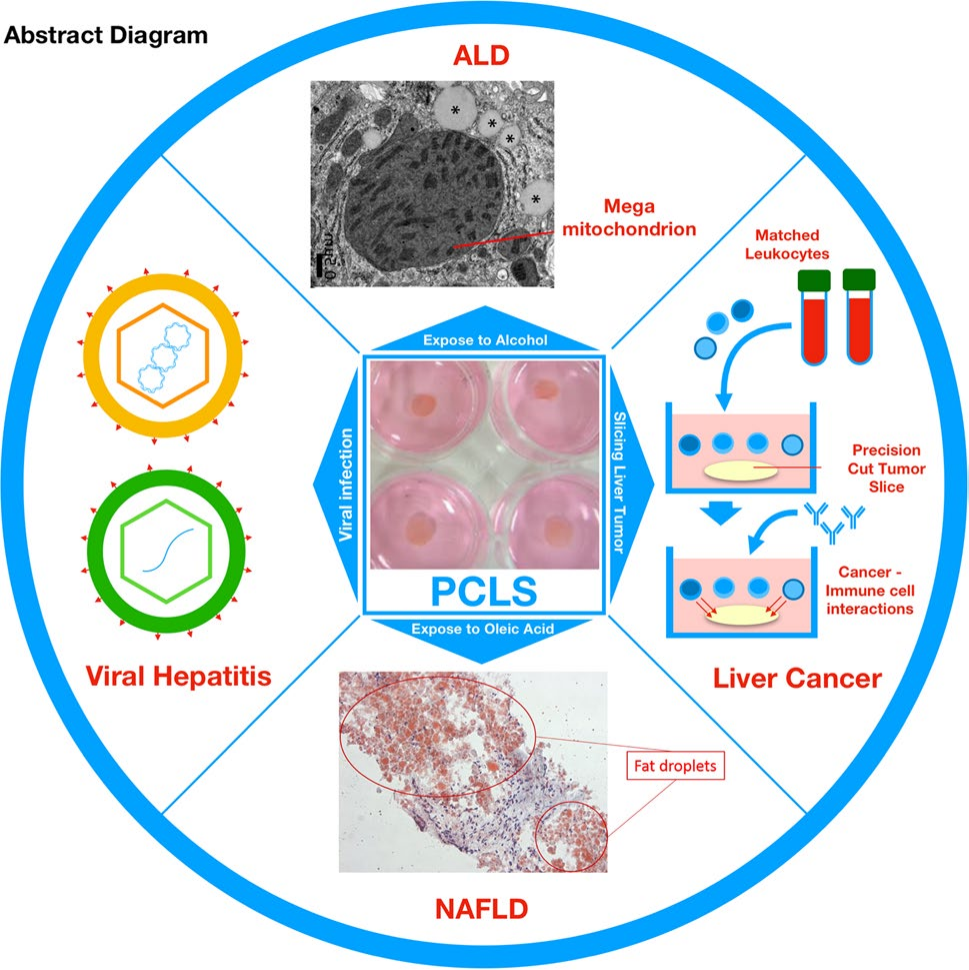

## Introduction

There is an urgent need for reliable systems to understand the pathogenesis of liver diseases, such as viral hepatitis, NASH, cancer, fibrosis and alcohol-induced liver disease. The in vitro cellular models and in vivo animal models currently utilised in liver research do not fully mimic the complex pathogenesis of liver injury and disease progression, particularly the development of fibrosis that occurs in humans. Lack of this understanding hinders the development of biomarkers both prognostic and diagnostic. Moreover, the pre-clinical testing of potential therapeutic targets in animal models can result in the identification of molecules that do not always show efficacy or worse still provoke toxic events in humans [1]. Recapitulating liver pathophysiology is challenging because of the intricacy of cellular composition and cell heterogeneity, the active role of the extracellular matrix and the complex tissue arrangement. Furthermore, immunity in man varies significantly from mouse models in terms of composition, strength and breadth, which is a problem that has to be addressed as inflammation and dysfunctional host immunity are major drivers of liver injury and disease.

**Fig. 1:**
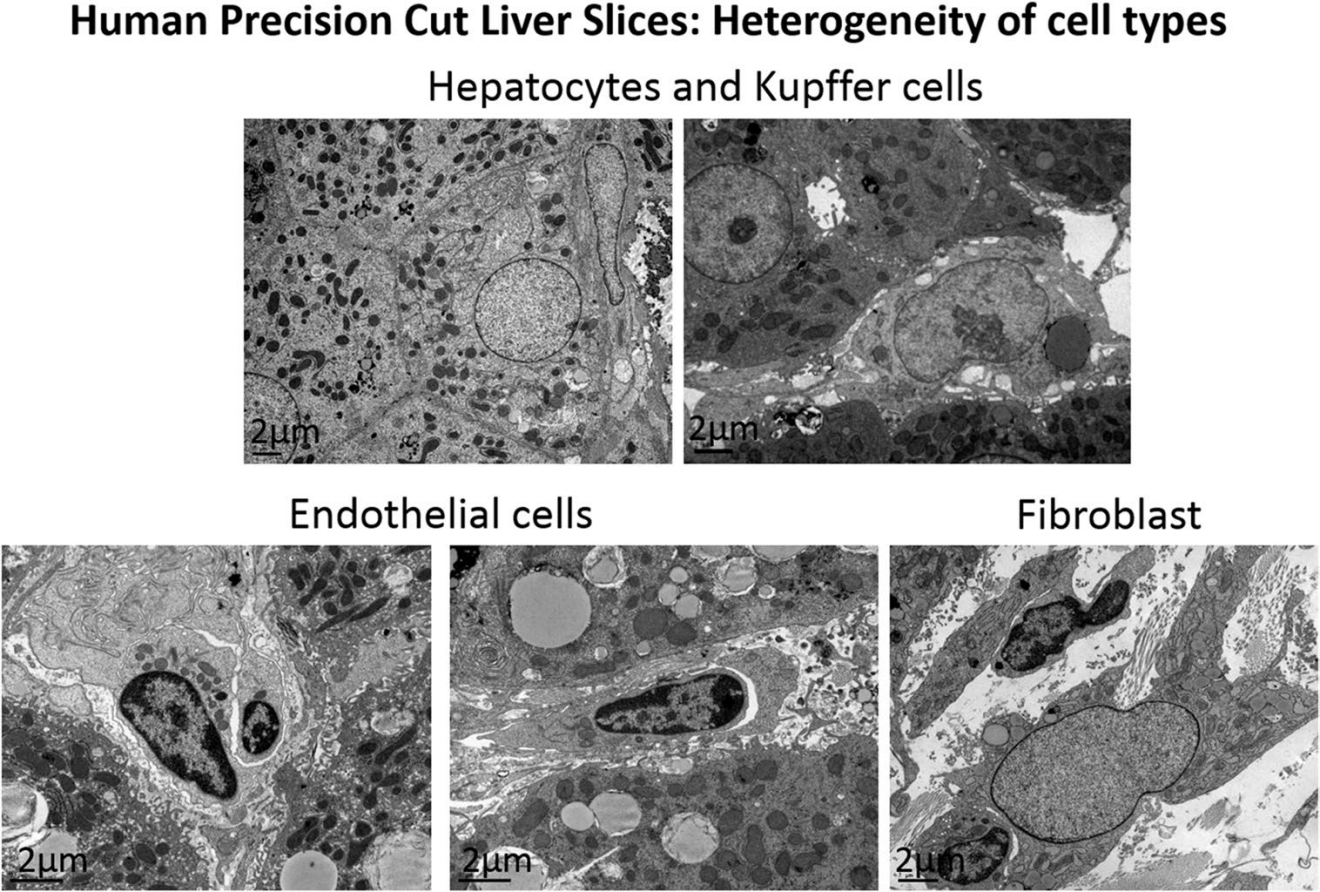
Cell heterogeneity in human Precision-Cut Liver Slices. Multiple cell types can be visualised in liver slices by electron microscopy, such as hepatocytes, kupffer cells, endothelial cells and fibroblasts

**Fig. 2:**
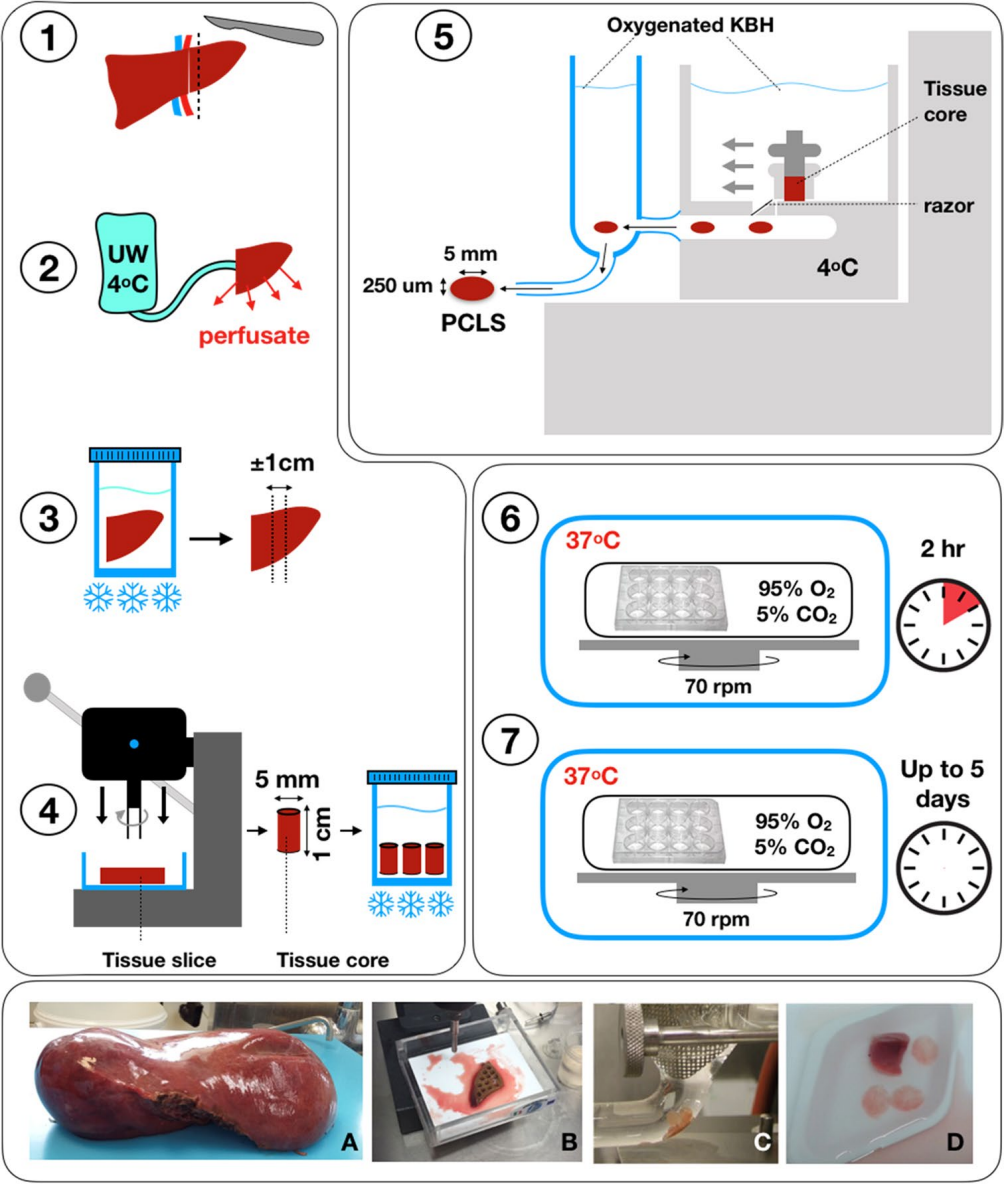
Precision Cut Liver Slices procedure. Resected liver tissue (1A) must be flushed immediately with ice-cold University of Wisconsin (UW) solution to remove blood (2). The flushed tissue sample is kept in sterile University of Wisconsin solution to reduce ischaemic damage and should be sliced within 3 h. A piece of approximately 1 cm thick is prepared from the resected tissue (3B). A hollow cylindrical tissue drill is used to prepare cores of 5 mm in diameter (Alabama R&D) (4B). The cores are inserted in the tissue core holder and cut with the tissue microtome (Krumdieck Tissue Slicer). Slices should have a wet weight of circa 5 mg and are approximately 250 µm thick corresponding to 10–15 cellular layers. This step is performed at 4 °C in Krebs–Henseleit buffer (KBH) saturated with carbogen (95%O_2_/5%CO_2_) (5C/D). After collection, each slice is cultured in supplemented Williams medium E with 5% human AB serum (full protocol details can be found in [3]). The medium is also saturated with carbogen and the plates kept in sealed chambers at 37 °C in an orbital shaker incubator (6). After an initial pre-incubation of 2 h to allow recovery, each slice is maintained in culture for up to 5 days and challenged with different insults or stimuli (7). Recent studies also suggest the possibility for maintaining functional hPCLS for 15 days [2] and we are currently testing this together with alternative media, atmospheric oxygen levels and other dynamic cultures, such as microfluidic systems, in order to improve slice viability

We believe that many of these limitations are overcome by the utilisation of human precision-cut liver slices (hPCLS). This versatile ex vivo model retains the multi-cellular histoarchitecture of the hepatic environment including liver-infiltrating immune cells. The liver slices are also reproducible, low cost and maintain the viability of hepatocytes, kupffer, endothelial, and hepatic stellate cells (Fig. 1) for up to 5 days in standard cultures and recent reports suggest that this may be extended to 15 days under the right conditions [2]. De novo disease processes can be studied in slices from healthy hPCLS challenged with toxic stimuli such as alcohol or fat and/or infected with hepatotropic viruses. Tissue for PCLS preparation is usually obtained from partial hepatectomy, from surgical waste for discard, explanted tissue or non-transplantable tissue. Liver slices can also be produced from diseased livers, such as patients with severe fibrosis and cirrhosis. Resected malignant/metastatic liver tissue can also be used to obtain tumour slices. The protocol for preparation of hPCLS established in our laboratory is adapted from the methods pioneered by Groothuis’ group [3] and is relatively straightforward (Fig. 2). Further to this, when slices are cultured in combination with autologous peripheral blood mononuclear cells, we can re-create the immunological interactions between the immune system and the healthy or diseased liver.

The precision-cut slices of kidney, intestinal and lung tissues [4] have been very successfully used to study multiple multicellular pathological processes such as in fibrosis and as shown below, liver slices are now emerging as a versatile tool to understand disease pathways in several liver conditions.

## Alcoholic liver disease (ALD)

**Fig. 3:**
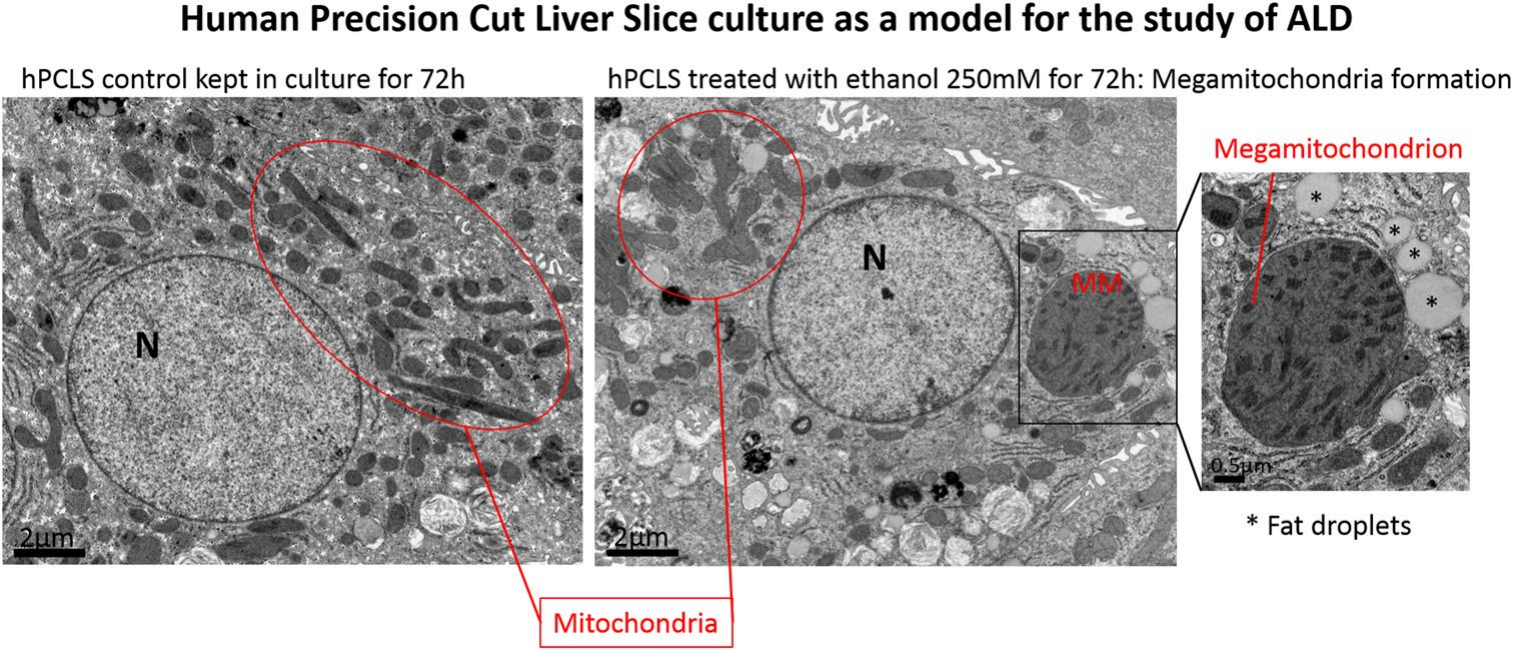
Development of megamitochondria in human Precision-Cut Liver Slices. Human liver slices were treated for 72 h with ethanol 250 mM and enlarged megamitochondria detected in the hepatocytes by electron microscopy. The shape and size of these mitochondria resemble organelles observed in liver biopsies from patients with Alcohol-induced Liver Diseases

ALD is associated with a wide spectrum of pathological manifestations including alcohol-related steatohepatitis, inflammation, fibrosis and cirrhosis. There is a paucity of models that mirror the pathogenesis of ALD and indeed it is particularly difficult to induce in rodents. Mice have a total aversion to alcohol and rapid alcohol metabolism prevents high blood alcohol levels. Even in animals continuously and chronically fed with alcohol by intragastric infusion, severe liver fibrosis does not develop, arguing for a different susceptibility to alcohol toxicity between animals and humans. Exposure of hPCLS to alcohol does induce liver injury, development of steatosis and oxidative stress; moreover, ethanol metabolism is preserved for days [5]. We have employed the hPCLS to investigate the impact of ethanol on the morphology and dynamics of mitochondria and we have shown that exposures for 24–72 h to doses ranging from 50 to 250 mM of ethanol induces the classical histological motifs of ALD, namely the development of disproportionately enlarged mitochondria in the hepatocytes [6] (Fig. 3). The presence of megamitochondria in ALD has been described since the 1970s and more recently their presence in liver biopsies has been interestingly associated with a lower risk of death at 90 days in patients with Alcoholic Hepatitis. However, to date, it has been very difficult to develop experimental models of alcohol-induced megamitochondrial development and, therefore, it is not understood whether they are actively involved in ‘protection’ from alcoholic insults and what mechanisms and pathways are involved in their appearance. Moreover, there is no data regarding their function and involvement in the pathogenesis of ALD. Our findings show that the hPCLS model is a novel model for (mega)mitochondria research and other changes in intracellular compartments and organelles.

## Non-alcoholic fatty liver disease (NAFLD)

**Fig. 4:**
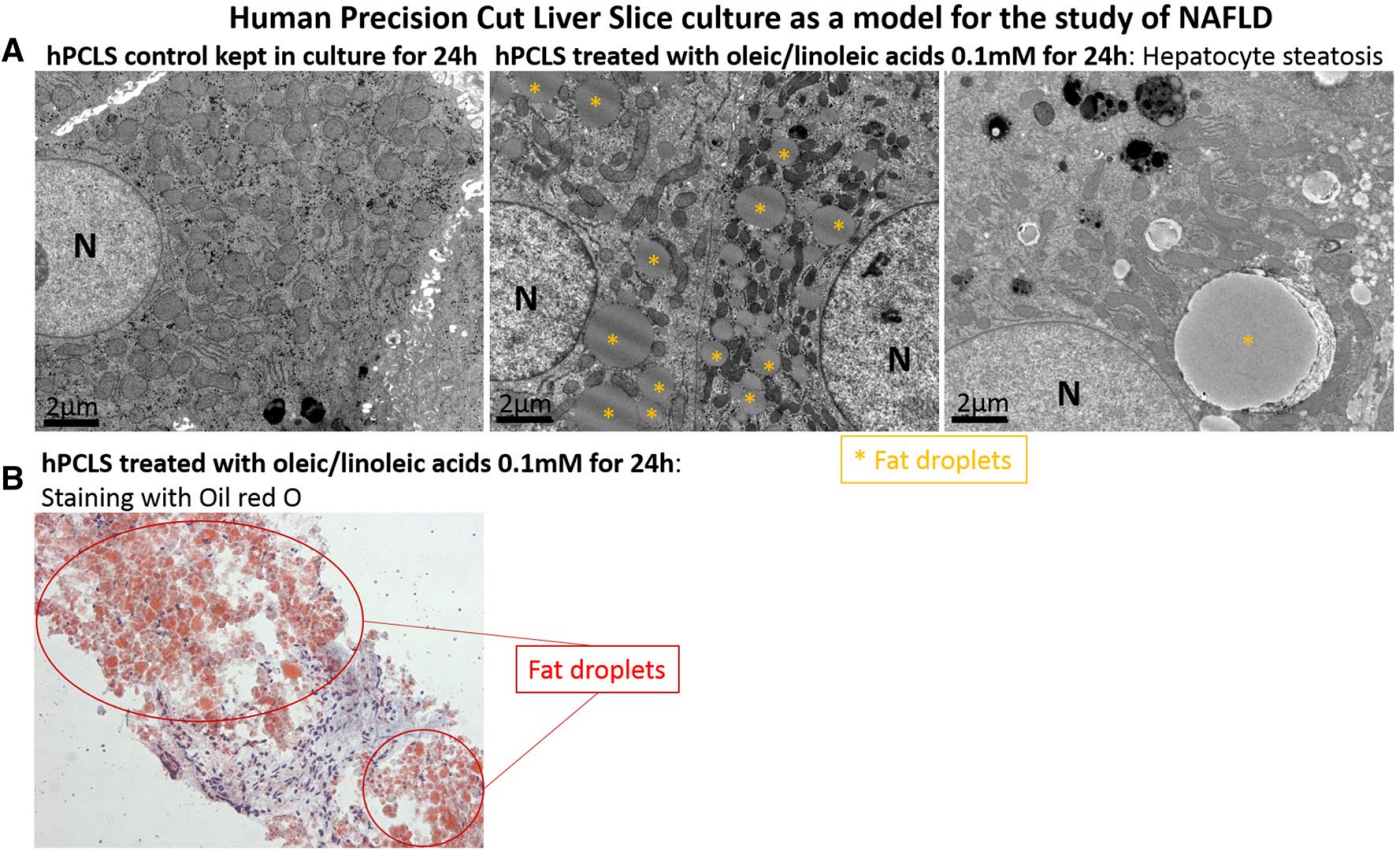
Fat accumulation in human precision-cut liver slices. Liver slices were exposed to a mixture of free fatty acids (oleic and linoleic acids 0.1 mM) for 24 h and fat accumulation was observed in the hepatocytes by electron microscopy (**a**) or by light microscopy after oil red-O staining (**b**). Different sizes of lipid droplets representing micro and macro-vesicular steatosis were recognised

In NAFLD, the presence of aberrant intrahepatic inflammation is associated with a significantly increased risk of liver injury and advanced chronic liver disease. The liver pathology that occurs in this specific metabolic and immunological context is not well recapitulated in mouse or rat models. Human PCLS which retain liver-infiltrating lymphocytes are a good model to study the changes observed during the different stages of NAFLD and test the efficacy of new drug compounds. We have treated hPCLS with free fatty acids (a mixture of oleic and linoleic acids, 0.1 mM) and we were able to mimic the hepatic steatosis, lipid deposition and lipotoxicity observed in patients during the early stages of NAFLD progression (Fig. 4). This model is also being used to understand whether we can protect from intrahepatic lipid accumulation through epigenetic manipulation.

Alternatively, use of liver explants from patients with NASH cirrhosis undergoing transplantation would help to assess if there was an opportunity for therapeutic interventions at this late stage of disease or whether the reversibility is restricted to earlier stages.

## Fibrosis and cirrhosis

There is an increasing evidence that different chronic liver diseases are characterised by different pro-fibrogenic mechanisms and while cirrhosis is the common result of progressive fibrogenesis, there are distinct patterns of fibrosis development associated with different etiologies. The induction of fibrosis from toxic agents can be demonstrated in the hPCLS, as the key events and the cellular components of fibrogenesis are maintained and remain active. Within each slice, the hepatocytes are metabolically fully competent and the other cell types retain their functionality including the inflammatory response induced by Kupffer cells and the development of fibrosis mediated by the activation of hepatic stellate cells and myofibroblasts. We have previously used mouse PCLS model to induce fibrogenesis with leptin and assess the ability of Osteopontin neutralisation to abrogate this response [7] confirming its utility as a pre-clinical model for anti-fibrotic therapies. Recent evidence suggests, however, that fibrogenic processes may be different in rodents. A recent study assessing the anti-fibrotic effects of LY2109761, a TGF-Beta inhibitor using human and rat PCLS showed that whilst LY2109761 was anti-fibrotic in both rat and human PCLS, there were considerable differences between the regulation of alpha-SMA and HSP47 in the responses to the treatment. Differences in fibrotic response between rat and human PCLS were also demonstrated earlier by the same group, demonstrating the strength of the PCLS model in fibrotic research while underlining the inter-species differences in mechanisms of fibrosis [8, 9].

Due to the versatility of the PCLS model, slices can also be prepared from diseased explants from patients with cirrhosis undergoing transplantation to allow the study of more advanced stages. An example is the study performed by Guyot et al. using ‘cirrhotic’ slices shows the suitability of this model to follow the processes of tissue remodelling by myofibroblasts and other hepatic cell types, allowing the comparison of cell behaviours between different pathological situations [10].

## Viral hepatitis

The hPCLS system has also been shown to have considerable utility in research studies in viral hepatitis. Liver slices are the ideal ex vivo model to investigate the mechanisms of viral infection and replication, viral life cycle and dynamics of virus propagation in native tissue and to evaluate the efficacy of new antiviral drugs. The infection of slices with hepatitis C virus (HCV) has been achieved using primary viral isolates from HCV-infected patient serum with high viral load not treated with antiviral drugs or with cell culture-grown viral strains of different genotypes (2a, 1a, 1b). In the study by Lagaye et al. the slice culture has been followed for up to 10 days and intracellular replication of HCV RNA, expression of viral proteins, extracellular production of infective particles demonstrated. Moreover, the slice supernatants of the cultured slices after infection were also able to further infect other liver slices [11]. In our laboratory we are currently developing the protocol for the infection of hPCLS with hepatitis B virus isolated from HepG2.2.15 cell culture and encouraging results have been achieved including cccDNA production (unpublished data). The infection of liver slices can also be performed using other types of viruses and we have tested both adenoviruses and adeno-associated viruses with consistent results. Genetic manipulation of the slices opens further the applicability of this powerful tool.

## Cholestatic disease and drug-induced liver injury

Human PCLS have been shown to produce bile acids and to be sensitive to drug-induced cholestasis [12]. Their capability of taking up bile acids or to express genes involved in bile acid transport and metabolism in a physiological and stable level validates the model for research in other important areas of hepatology, including drug-induced liver injury (DILI). Understanding the mechanisms of DILI is a major challenge to contemporary hepatology. DILI accounts for more than 50% of the cases of acute hepatic failure and is associated with a case-fatality rate of 10–50% depending on the drug involved. Hepatic drug reactions are difficult to diagnose as they can mimic hepatobiliary disease which makes distinguishing DILI and spontaneous hepatic disease difficult. The currently available diagnostic tests for DILI report on dying cells and signal the presence of injury after considerable damage has taken place. The hPCLS could be used to provide detailed insight into molecular mechanisms underlying the toxic endpoints of DILI to identify early signatures of liver-specific toxicity.

## Hepatocellular carcinoma (HCC)

**Fig. 5:**
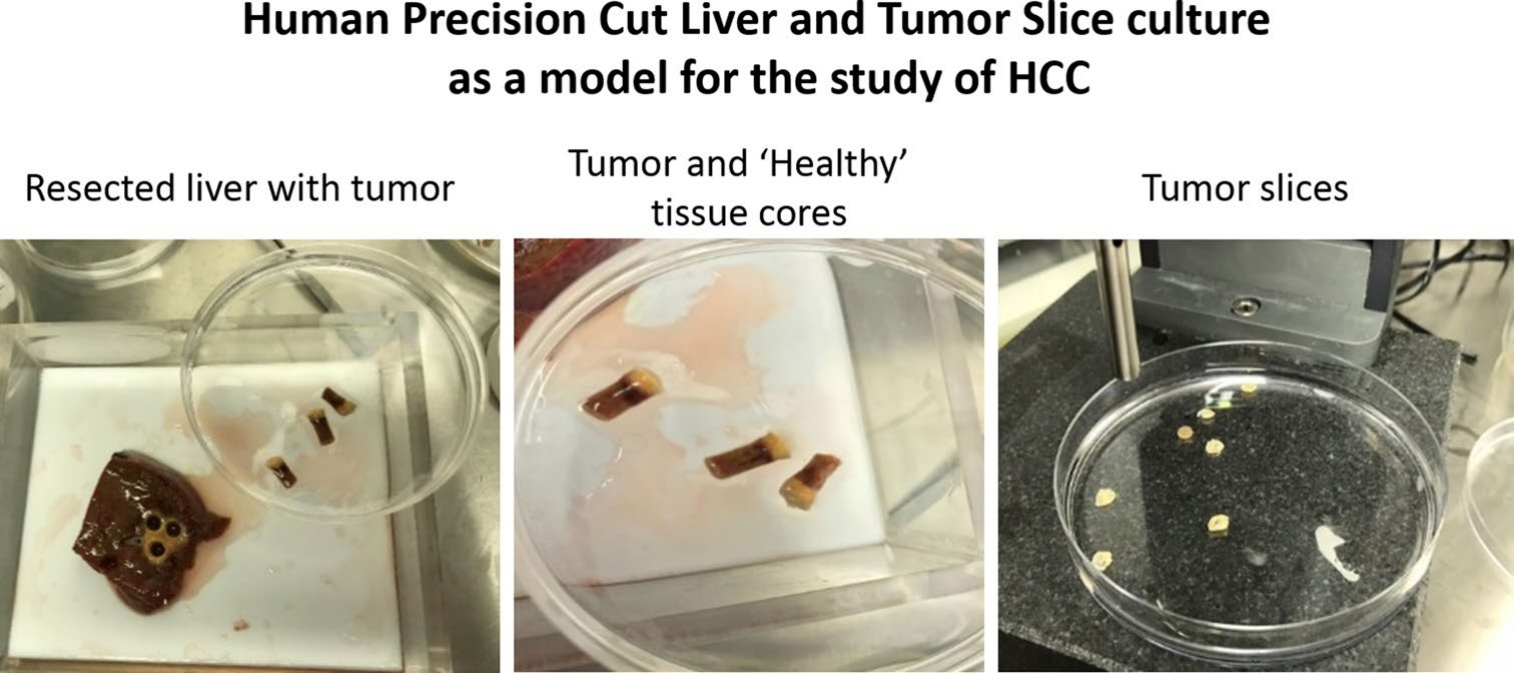
Human precision-cut liver and tumour slices. Tissue cores were drilled from resected liver/tumour tissue using the Tissue Coring Press by Alabama-R&D. Cores included both non-tumorigenic and tumour tissue. The tumour slices obtained can be then utilised for the study of molecular pathways during HCC or for testing of antineoplastic drugs

Hepatocellular carcinoma is the fifth most common cancer globally and the second leading cause of cancer-related mortality worldwide. Many cytotoxic chemotherapeutic agents have been tested in patients with advanced disease with disappointing outcomes and poor tolerance. Sorafenib was the first systemic therapy approved for HCC; however, it is associated with significant toxicity and low response rates. Tissue slices from HCC (HCC-hPCLS) retain the tumor microenvironment and have been shown to represent a valuable system for pre-clinical testing of anti-HCC drugs [13, 14] (Fig. 5). To date, sorafenib has not been tested on HCC-hPCLS, although it has been used on PCLS in the context of liver fibrosis along with several other Tyrosine Kinase Inhibitors [9]. We believe this model could be a strong tool in predicting patient-specific responses and drug resistance to approved and novel cytostatic compounds. The tumor slices can be utilised to evaluate drug-induced pro-apoptotic effects by histopathological analysis and to investigate side effects of anti-cancer therapies with off-target effects on the surrounding healthy/cirrhotic tissue from the same donor. Experiments can also be run in concert in liver slices that are cut across the tumor margin, i.e. a single slice would have both tumor and neighbouring portions.

Besides antineoplastic drugs, oncolytic viruses have been successfully tested on hPCLS derived from HCC tumors. Zimmerman et al. [14] used the slice model as a predictive test for patient-specific tumor response and to evaluate the safety of an oncolytic vaccine viruses. Given the importance of the immune system in HCC [15], the development of potential immunotherapeutic options such as checkpoint inhibitors for HCC will be accelerated by availability of relevant immunocompetent models of HCC to test the rationale of combination therapies, early identification of biomarkers and designing personalised approaches. The use of hPCLS co-cultured with matched peripheral leukocytes may hold the key for assessing cancer-immune cell interactions and this is an area of current research interest for the hPCLS system.

## Limitations

Local access to freshly resected (human) tissue is one of the main limitations of this technique; the procedure of acquiring fresh tissue samples requires co-ordination and good communication between the stakeholders, including surgeons, histopathologists, nurses and researchers.

Further to this, the ‘healthy’ tissue taken from resected samples is not truly healthy, as it is inevitably from the distal portion of a diseased liver. This can bring variability between experiments and subjects. The same applies to the use of diseased (cirrhotic/HCC) slices, as oftentimes these patients have previously undergone multiple different (chemo)therapeutic/ablative strategies prior to surgery. This can not only add inconsistency between samples but also compromise the physical (structural integrity/fat content) and physiological properties of the tissue. Other personalised approaches do not suffer from these drawbacks such as the use of induced pluripotent stem cells (iPSC) which are flexible and feasible for personalised medicine, but it must be stressed that iPSC lack the original cellular and genetic landscape of the diseased tissue.

## Final remarks

Chronic liver disease and cirrhosis are a major cause of illness and death worldwide and this is projected to substantially increase in the future. Considering NAFLD alone, forecasts predict an exponential rise in disease burden from 83.1 million in 2015 to 100.9 million by 2030. Primary liver cancer rates are also increasing and HCC is currently the second leading cause of global cancer mortality. As such, there are multiple needs for models of liver disorders to study prognostic and diagnostic markers and for drug discovery.

A (hu)man is not a big mouse and inter-species differences in disease processes and immunity amplify small differences in complex pathological pathways and drug responsiveness. Preservation of the original 3D architecture and the lobular structure of the liver in the human precision-cut liver slices provide the essential requirement for a good pre-clinical model and use of this technique will increase our understanding of the pathophysiology of different liver diseases.
